# Genome-Wide Contribution of Genotype by Environment Interaction to Variation of Diabetes-Related Traits

**DOI:** 10.1371/journal.pone.0077442

**Published:** 2013-10-28

**Authors:** Ju-Sheng Zheng, Donna K. Arnett, Yu-Chi Lee, Jian Shen, Laurence D. Parnell, Caren E. Smith, Kris Richardson, Duo Li, Ingrid B. Borecki, José M. Ordovás, Chao-Qiang Lai

**Affiliations:** 1 Department of Food Science and Nutrition, Zhejiang University, Hangzhou, China; 2 Jean Mayer USDA Human Nutrition Research Center on Aging at Tufts University, Boston, Massachusetts, United States of America; 3 Department of Epidemiology, University of Alabama at Birmingham, Birmingham, Alabama, United States of America; 4 Bone & Mineral Unit, Division of Endocrinology, Oregon Health & Science University, Portland, Oregon, United States of America; 5 Department of Genetics, Washington University School of Medicine, St. Louis, Missouri, United States of America; CAEBi, Spain

## Abstract

While genome-wide association studies (GWAS) and candidate gene approaches have identified many genetic variants that contribute to disease risk as main effects, the impact of genotype by environment (GxE) interactions remains rather under-surveyed. To explore the importance of GxE interactions for diabetes-related traits, a tool for Genome-wide Complex Trait Analysis (GCTA) was used to examine GxE variance contribution of 15 macronutrients and lifestyle to the total phenotypic variance of diabetes-related traits at the genome-wide level in a European American population. GCTA identified two key environmental factors making significant contributions to the GxE variance for diabetes-related traits: carbohydrate for fasting insulin (25.1% of total variance, *P*-nominal = 0.032) and homeostasis model assessment of insulin resistance (HOMA-IR) (24.2% of total variance, *P*-nominal = 0.035), n-6 polyunsaturated fatty acid (PUFA) for HOMA-β-cell-function (39.0% of total variance, *P*-nominal = 0.005). To demonstrate and support the results from GCTA, a GxE GWAS was conducted with each of the significant dietary factors and a control E factor (dietary protein), which contributed a non-significant GxE variance. We observed that GxE GWAS for the environmental factor contributing a significant GxE variance yielded more significant SNPs than the control factor. For each trait, we selected all significant SNPs produced from GxE GWAS, and conducted anew the GCTA to estimate the variance they contributed. We noted the variance contributed by these SNPs is higher than that of the control. In conclusion, we utilized a novel method that demonstrates the importance of genome-wide GxE interactions in explaining the variance of diabetes-related traits.

## Introduction

Type 2 diabetes (T2D) is one of the most common chronic diseases in the world, accounting for nearly 90% to 95% of all diabetes cases. Approximately 25.6 million adults in the U.S.A. [1] and 285 million adults worldwide [2] were affected by diabetes in 2010, and it is estimated that between 2010 and 2030, the number of adult diabetes cases will increase by 69% in developing countries and by 20% in developed countries [2]. For the prevention of T2D, identifying genetic and environmental risk factors has been a primary research focus in the public health arena. Thus far, more than 100 single-nucleotide polymorphisms (SNP) for T2D and T2D-related traits have been identified via genome-wide association studies (GWAS) (Hindorff et al. www.genome.gov/gwastudies). However, the GWAS-identified genetic variants explain only about 10% of T2D heritability [3], [4]. The “missing heritability” may be attributed to variants of small effect, rare variants, structural variants poorly captured by GWAS arrays, copy number variants, weak linkage disequilibrium of genotype variants with the causal variants, gene-gene interaction, and genotype by environment (GxE) interaction [5], [6]. Because GxE interactions suggest a way by which genetic risk may be ameliorated, these environmental factors are of great relevance to public health, and are the focus of a growing number of studies [7].

Environmental factors, such as diet and lifestyle, are important in the onset, development and progression of T2D and its related phenotypes [8], [9]. The interactions of environmental factors with genotypes contribute to the total genetic variance of a given trait [10], and are important constituents of the total phenotypic variance. While a number of studies have demonstrated the significant effects of GxE on T2D and T2D-related traits [7], [11], a further clarification of the role of GxE at the genome-wide level could help predict disease risk more accurately and help develop dietary recommendations to improve prevention and treatment. In addition, for T2D-related traits, such as insulin resistance and pancreatic β-cell function, there are still no published data examining to what extent variance of these traits are explained by the GxE interaction at the genome-wide level. It is crucial to estimate the proportion of GxE interaction variance for T2D-related traits in addition to the main effect of the genetic variants because this produces a more complete understanding of the role of environment with regard to these phenotypes. Furthermore, it is of profound interest to understand which dietary or lifestyle factors are the most influential for the variation of a given T2D-related phenotype through GxE interactions and to what extent these environmental factors contribute to the phenotypic variation. In this study, we aimed to explore the variance contribution of GxE interactions to four T2D-related traits at the genome-wide level in a population of European ancestry living in the U.S.A.

## Research Design and Methods

### Study Population

A total of 820 subjects (406 men and 414 women) participating in the Genetics of Lipid Lowering Drugs and Diet Network (GOLDN) Study were included in the present study. All participants were of European origin and re-recruited from three-generational pedigrees in the two centers of the National Heart, Lung, and Blood Institute Family Heart Study in Minneapolis, MN, and Salt Lake City, UT. Details of the study design and methodology for GOLDN were described [12]. The T2D-related traits from the second visit at the baseline were used for the analysis of this study. The study protocol was approved by the Institutional Review Boards at the University of Minnesota, University of Utah, and Tufts University. All participants gave written informed consent.

### Genome-wide Genotyping

Extraction and purification of genomic DNA have been described [13]. Genome-wide genotyping was conducted by the Affymetrix Genome-Wide Human SNP Array 6.0 (CA, USA) and Birdseed calling algorithm [14], and 906,600 SNPs were genotyped. A total of 590,000 SNPs among those genotyped SNPs were selected for our genome-wide analysis after they met the following criteria: minor allele frequency ≥5%, call rate ≥96% and *P*-value ≥1.0E-6 for the Hardy-Weinberg equilibrium (HWE) test, and there were negligible Mendelian errors within family [15].

### Determination of Dietary and Lifestyle Factors and T2D-related Traits

A diet history questionnaire (DHQ) developed by National Cancer Institute was used to assess dietary intake, and nutrient intake was then estimated based on the DHQ and the national dietary data (USDA Continuing Survey of Food Intakes by Individuals) [16]. The DHQ has been validated in two studies [17], [18]. A questionnaire was used to assess lifestyle information. A total of 15 dietary and lifestyle factors (**Table 1**) possibly related to T2D-related traits based on the literature and our experience were used for the GxE analysis. There were 12 dietary factors: glycemic load, protein, total fat, saturated fat, monounsaturated fat (MUFA), polyunsaturated fatty acid (PUFA), n-3 PUFA, n-6 PUFA, n-3: n-6 PUFA ratio, carbohydrate, fiber and trans-fat, and three lifestyle factors: alcohol use, smoking status, and physical activity. All the dietary intakes (except glycemic load) and alcohol use were expressed as percentage of total energy intake and categorized into quartiles for data analysis. Physical activity and glycemic load were also categorized into quartiles, while smoking status was grouped into three groups: non-smoker, past smoker and current smoker.

**Table 1:** Demographic and biochemical characteristics and dietary and lifestyle data in the GOLDN population^1^.

	Men (n = 406)		Women (n = 414)	
	Mean ± SD	Range (Q1–Q3)	Mean ± SD	Range (Q1–Q3)
Age, y	48.8±15.9	38.0–62.0	49.0±16.1	39.0–62.0
BMI, kg/m^2^	28.6±4.7	25.8–31.1	28.4±6.2	23.8–31.7
Fasting glucose (mg/dL)	105.8±21.5	96.0–108.0	98.3±17.0	90.0–101.0
Fasting insulin (mU/L)	14.56±8.36	9.0–17.0	13.6±8.1	9.0–16.0
HOMA-IR	1.93±1.09	1.22–2.25	1.78±1.1	1.15–2.11
HOMA-B, (%)	108.8±38.8	83.9–130.5	116.6±36.3	91.7–135.6
Current smoker, n (%)	33 (8.1)		34 (8.2)	
Current drinker, n (%)	199 (49.0)		208 (50.2)	
Physical activity score	34.9±7.3	30.3–38.2	33.1±5.0	29.8–35.3
Glycemic load	145.4±86.2	92.8–174.5	108.8±55.7	74.5–128.3
Total energy (kcal/day)	2505±1501	1669–2993	1781±817	1286–2099
Protein (% of total energy)	15.8±2.7	14.1–17.5	15.8±2.8	14.2–17.5
Total fat (% of total energy)	35.9±6.7	31.5–40.3	35.1±6.9	30.4–39.7
Saturated fat(% of total energy)	12.1±2.7	10.5–13.9	11.5±2.6	9.67–13.0
MUFA (% of total energy)	13.7±2.8	11.9–15.4	13.0±2.8	11.0–14.9
PUFA (% of total energy)	7.39±1.99	6.05–8.41	7.95±2.34	6.25–9.38
n-3 PUFA (% of total energy)	0.68±0.19	0.54–0.79	0.75±0.23	0.58–0.87
n-6 PUFA (% of total energy)	6.64±1.83	5.45–7.61	7.14±2.16	5.56–8.42
n-3: n-6 PUFA (% of total energy)	0.10±0.02	0.09–0.12	0.11±0.02	0.10–0.12
Carbohydrate (% of total energy)	47.5±8.6	41.7–53.5	50.3±8.1	45.0–55.4
Alcohol use (% of total energy)	2.78±6.57	0.01–2.41	1.35±3.35	0.01–1.14
Trans fat (% of total energy)	2.20±0.58	1.82–2.50	2.11±0.66	1.68–2.46
Fiber (% of total energy)	1.82±0.48	1.49–2.06	2.09±0.62	1.65–2.48

1 Values are mean ± SD or n (%). BMI, body mass index; SD, standard deviation; Q, quartile.

Fasting glucose was measured by a hexokinase-mediated reaction on the Hitachi commercial kit (Linco Research, St. Charles, MO), fasting insulin was determined by a commercial kit by radioimmunoassay (Linco Research, St. Charles, MO). Homeostasis model assessment of insulin resistance (HOMA-IR) and of β-cell function (HOMA-B) were estimated by Levy’s computer model [19]. HOMA-IR, insulin and glucose were Box-Cox transformed [20] to achieve normal distribution before analysis.

### Estimation of Variance Contribution by GxE Interaction using GCTA

A tool for Genome-wide Complex Trait Analysis (GCTA) [21] was used to assess the contribution of GxE interaction to the phenotypic variations of T2D-related traits for each of the 15 dietary and lifestyle factors. Currently, GCTA is suitable for the variance estimation only of continuous variables, and estimation based on binary variables, such as disease status, are not possible. By using a -gxe option, the GxE interaction effects were treated as random effects in the model, while the main effects of the genetic variants and environmental factors were treated as fixed effects. Covariates in the model included age, sex, study center, kinship and population structure. Population structure was estimated based on principle component analysis using SVS (Golden Helix Inc., Bozeman, MT.) [22], [23], and three key principle components were selected as covariates in the analysis. Heritability of GxE for T2D-related traits was estimated as the GxE variance divided by the total phenotypic variance. The main steps of running GCTA in a Linux computer environment include: 1) generate bed, bim and fam files for GWAS genotype data using PLINK; 2) generate grm.gz and grm.id files using “–make-grm”; 3) prepare a phenotype file for each trait and a covariate file; 4) estimate the GxE variance contribution by introducing a “-gxe” option.

### GCTA Bootstrap Analysis

A bootstrap analysis was performed to determine if the significant GCTA heritability estimates obtained from SNPs identified in each GxE GWAS could be obtained by chance. This was done for Insulin × Carbohydrate, HOMA-IR × Carbohydrate, and HOMA-B × Carbohydrate.

A perl script was written to extract 1000 random sets of 49, 51 or 39 (numbers corresponding to SNPs identified in each significant GxE from above) SNPs from the GOLDN genotype data. A Unix script was then written to generate data for bootstrap analysis. For each of the 1000 sets of SNPs the script first created a GRM file and second used this GRM to perform a GxE analysis applying the GCTA parameters used in the original analysis. This script was run three times, once for each GxE. For each bootstrap analysis, the resulting heritability estimates and *P*-values were ranked and the original values compared against the 95^th^ percentile.

### GxE Genome-wide Association Study using GWAF

Linear mixed effects model (LME) was used to test the GxE interactions for T2D-related traits at the genome-wide level under an additive genetic model while adjusting for age, sex, study center, kinship and population structure. All SNP genotypes and interactions were treated as fixed effects, while family relationship was treated as a random effect through the kinship matrix in R (version 2.15.0, GWAF package) [24]. Quantile-quantile (Q-Q) plots of *P*-values were drawn using R.

### Correction for Multiple Testing

By using an online tool called MatSpD (http://gump.qimr.edu.au/general/daleN/matSpD/), we first calculated the number of independent variables represented by these 15 environmental factors and four diabetes-related traits to be 13 and three, respectively. Based on these numbers, we then corrected for multiple testing in GCTA analysis by applying Bonferroni correction and the corrected *P*-value for significance is 0.001 (0.05/(13×3)). For GWAS, a *P*-value <1.0E-5 was considered as statistically significant, as this is a commonly used a threshold for discovery in GWAS (http://www.genome.gov/gwastudies/).

## Results

### Variance Contribution of GxE Interaction for T2D-related Traits at the Genome-wide Level

**Figure 1:**
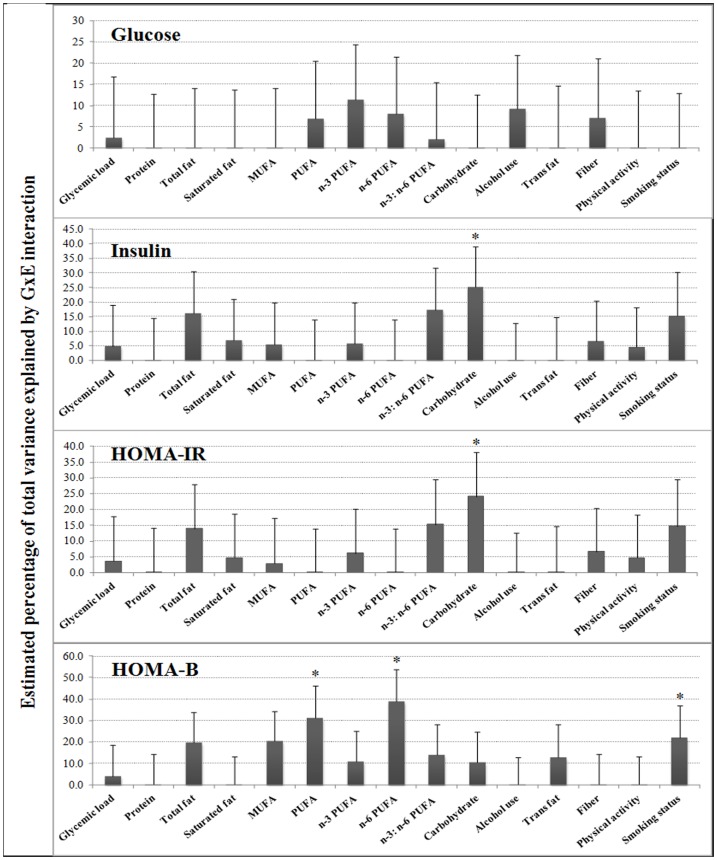
Estimation of GxE variance of 15 dietary and lifestyle factors for four diabetes-related traits. The GxE variance is shown as the percentage of the total phenotypic variance of each trait. *P<0.05 indicates nominal significant contribution to total variance.

GCTA [21] was used to estimate the contribution of genome-wide GxE variance to T2D-related traits while adjusting for potential confounders: age, sex, study center, and population structure. For fasting glucose, additive genetic variance contributed 19.8% (*P*- nominal = 0.002) of total phenotypic variance, but in this population, none of the variance from GxE interactions contributed significantly to the total glucose variance (**Figure 1**, **Table S1**). The results were similar when further adjusting for body mass index (BMI) and no significant GxE variance contribution was observed.

For fasting insulin, additive genetic variance accounted for 20.2% (*P*- nominal = 0.002) of total phenotypic variation. After inclusion of GxE in the model, the variance explained by the additive genetic variance varied from 10.9% to 19.5%, and carbohydrate intake contributed significant GxE variance to the total variance of fasting insulin (25.1%, *P*-nominal = 0.032) (**Table 2** **,** **Figure 1** **,** **2** **,** **3**). The GxE variance of dietary n-3: n-6 PUFA ratio, although not significant (*P*-nominal = 0.112), is substantial (17.4%) compared to those of the other dietary or lifestyle factors, for which GxE variances were not significant (*P*-nominal >0.05) (**Table S2**). Inclusion of BMI into the covariates only slightly changed the results, and the genome-wide variance contribution by carbohydrate intake was 29.1% (*P*-nominal = 0.021).

**Figure 2:**
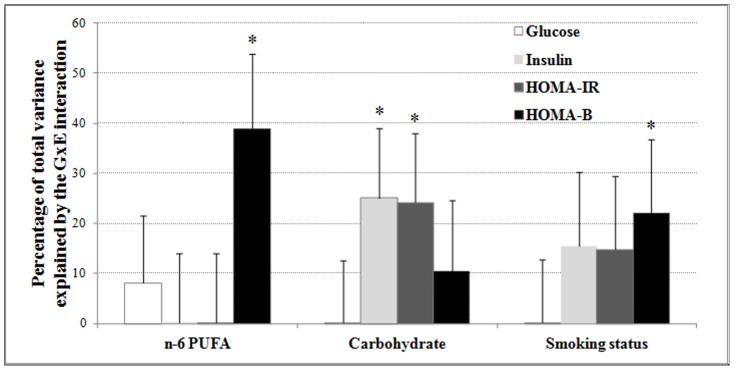
Estimated amount of variance by GxE interaction of three E factors for four diabetes traits. The GxE variance is shown as the percentage of the total phenotypic variance of each trait. *P<0.05 indicates nominal significant contribution to total variance.

**Figure 3:**
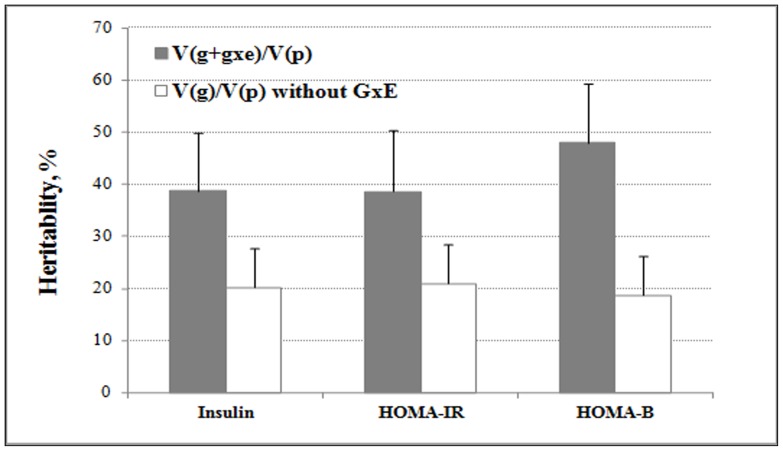
Estimated heritability (%) of type 2 diabetes-related traits. Unfilled bars depict the heritability based on additive genetic variance. Solid bars represent heritability, as a percentage, due to the sum of additive genetic variance and genetic variance by GxE interaction. The corresponding environmental factor for insulin, HOMA-IR and HOMA-B was carbohydrate, carbohydrate, and n-6 PUFA, respectively.

**Table 2:** Estimation of additive genetic variance and variance of GxE interaction for diabetes-related traits^1^.

Trait	E factor	Nominal *P*-value (gxe)	Vg	SE	Vgxe	SE	h^2^ (g), %	SE	h^2^ (gxe), %	SE	h^2^ (g+gxe), %
Fasting insulin^2^	Carbohydrate	0.032	0.00048	0.00031	0.00089	0.00050	13.6	8.6	25.1	14.0	38.7
HOMA-IR^2^	Carbohydrate	0.035	0.0013	0.0008	0.0021	0.0012	14.5	8.6	24.2	13.9	38.7
HOMA-B^3^	PUFA	0.016	148.7	102.7	370.0	175.4	12.6	8.6	31.4	14.6	44.0
	n-6 PUFA	0.005	105.5	104.5	459.8	180.4	8.9	8.8	39.0	14.9	48.0
	Smoking status	0.055	49.0	145.1	255.7	174.9	4.2	12.5	22.0	14.9	26.2

1 Vg, additive genetic variance; Vgxe, variance contributed by GxE interaction; SE, standard error; h^2^ (g), heritability; h^2^ (g + gxe), total heritability. Only the significant E factors are listed here, while the results of other E factors are in supplemental files.

2 GCTA was adjusted for age, sex, study center, kinship, and population structure.

3 GCTA was adjusted for age, sex, body mass index, study center, kinship, and population structure.

For HOMA-IR, the additive genetic variance accounted for 20.9% of total variance (*P*-nominal = 0.001). Similar to fasting insulin, GxE interaction of carbohydrate intake showed the most significant contribution (*P*-nominal = 0.035), accounting for 24.2% of total variance (**Table 2** **,** **Figure 1** **,** **2** **,** **3**). The GxE contribution of carbohydrate intake was similar when further adjusted for BMI (*P*-nominal = 0.02, accounting for 29.4% of total variance). None of the GxE interactions from other dietary or lifestyle factors contributed significantly to the total phenotypic variance of HOMA-IR (*P*-nominal >0.05) (**Figure 1** **, Table S3**).

For HOMA-B, significant differences of variance contributions were observed for most environmental factors before and after adjustment of BMI, therefore BMI was added into the model. We observed that 18.7% of the total variance could be explained by the additive genetic variance (*P*-nominal = 0.005). The most significant GxE variance was contributed by n-6 PUFA intake (39.0%, *P*-nominal = 0.005), while the GxE variance contributed by dietary PUFA was also significant (31.4%, *P*-nominal = 0.016) (**Table 2**, **Figure 1**). Another environmental factor that was a marginally significant contributor to the GxE variance was smoking status, which accounts for 22.0% of total HOMA-B variance (*P*-nominal = 0.055) (**Table 2**, **Table S4**).

As dietary or lifestyle factors were not totally independent from each other, we then examined whether the contribution to the total phenotypic variance from a significant GxE interaction was affected by other dietary or lifestyle factors. We approached this by pairing one significant environmental factor with another factor simultaneously in the model, while controlling for potential confounders (**Table S5**). For fasting insulin, we paired carbohydrate intake with n-3: n-6 PUFA ratio, total fat, and smoking status in the model respectively, and inclusion of either of these factors did not remarkably change the variance contributed by the GxE of carbohydrate intake (19.8%–23.6%). For HOMA-IR, similar to insulin, including n-3: n-6 PUFA ratio, total fat, or smoking status in the model did not significantly change the contribution of GxE variance from carbohydrate intake to the total variance, and the GxE contribution of carbohydrate varied from 19.6% to 22.6%. For HOMA-B, inclusion of n-6 PUFA and PUFA in the model abolished the GxE variance contributed by dietary PUFA, while the GxE variance contributed by n-6 PUFA did not change remarkably (45.5%). This was because of the strong correlation between n-6 PUFA and PUFA (r = 0.89, *P*<0.001) in this population and indicated that n-6 PUFA and PUFA represented the same GxE variance for HOMA-B. Pairing total fat, MUFA, and smoking status with n-6 PUFA in the model did not change the GxE variance estimate of n-6 PUFA.

### Influence of Major Environmental Factors on T2D-related Traits in GxE GWAS

Using GCTA, we identified environmental factors that showed significant GxE contributions to the variance of a given T2D-related trait. To illustrate and confirm the results from GCTA, a GxE GWAS was conducted. Selecting the most significant environmental factor for each trait, we used a GWAF package in R [24] to conduct a GxE GWAS, and a non-significant dietary or lifestyle factor was used as a control to run the analysis. A GWAS without GxE in the model was also conducted for each trait as a comparison to the GxE GWAS. The significant environmental factor for the GxE GWAS for insulin and HOMA-IR was carbohydrate intake, while n-6 PUFA was chosen for HOMA-B. Dietary protein intake did not contribute to the GxE for any T2D-related trait and therefore served as a control for all traits. We observed that, for those environmental factors with significant GxE contribution to the phenotypic variance, the QQ-plots were slightly off the diagonal, while for protein intake, the QQ-plots aligned well with the diagonal compared to the significant environmental factors (**Figure S1, S2, S3**).

We extracted all SNPs from each GWAS with a nominal *P*-value <1.0E-5 for the main effect and for GxE interaction for insulin, HOMA-IR and HOMA-B (**Table 3**). Detailed information for the resulting 119 SNPs is provided in **Table S6**. The number of SNPs (nominal *P*-value for GxE <1.0E-5) for the GxE GWAS of carbohydrate was much larger than that of protein for both insulin (28 SNPs vs 6) and HOMA-IR (27 SNPs vs 6). For HOMA-B, GxE GWAS of n-6 PUFA produced a larger number of SNPs (nominal *P*-value for GxE <1.0E-5) compared with protein control (26 SNPs vs 2).

**Table 3:** Number of SNPs with nominal *P*-value ≤10E-5 based on GxE GWAS.

Trait	E factor	GxE^1^	Without GxE	With GxE in the model		
				Main effect	GxE interaction	Sum of main effect and GxE
Insulin^2^	Carbohydrate	Significant	8	21	28	37
	Protein	Non-significant	7	6	6	12
HOMA-IR^2^	Carbohydrate	Significant	9	22	27	38
	Protein	Non-significant	7	7	6	13
HOMA-B^3^	n-6 PUFA	Significant	14	15	26	37
	Protein	Non-significant	17	0	2	2

1 The E factor has a significant or non-significant GxE variance contribution to the total phenotypic variance.

2 GWAS adjusted for age, sex, study center, kinship, and population structure.

3 GWAS adjusted for age, sex, body mass index, study center, kinship, and population structure.

### Estimation of GxE Variance Explained by the Variants Identified from the GxE GWAS

For each trait (insulin, HOMA-IR and HOMA-B), the SNPs with a nominal *P*-value <1.0E-5 for the main effect or GxE effect from the corresponding GxE GWAS were extracted for the estimate of variance contribution of these SNPs to the total phenotypic variance using GCTA (**Table 4**). Both significant and non-significant (dietary protein) environmental factors were included in the model. For HOMA-IR, the additive genetic variance explained by the 51 SNPs accounted for only 3.2% of the total variance, while surprisingly, the GxE interaction of carbohydrate intake represented up to 27.8% of the total variance (*P*-nominal = 6.35E-25). However, the GxE interaction with protein intake accounted for just 7.5% of the total HOMA-IR variance (*P*-nominal = 6.90E-4). Similarly, GxE interaction of 49 SNPs with carbohydrate intake explained 28.6% of total insulin variance (*P*-nominal = 1.11E-23), while the GxE for protein explained 7.5% variance of the trait (*P*-nominal = 5.82E-4), and the additive genetic variance was negligible. For HOMA-B, the GxE of n-6 PUFA represented 23.3% of total variance of the trait (*P*-nominal = 1.68E-22), while the variance explained by the GxE of protein contributed only 1.4% of the total variance (*P*-nominal = 0.179). Bootstrap analysis showed that our GCTA GxE *P*-value falls far below the 95th percentile indicating that these results are highly unlikely to be obtained merely by chance (**Table 4**).

**Table 4:** Estimation of heritability (%) from identified SNPs with nominal *P*-value <1.0E-5 based on GxE GWAS^1^.

Trait	E factor	#SNP	h^2^ (g), %	SE	h^2^ (gxe), %	SE	Nominal*P*-value	Bootstrapping 95thpercentile *P*-value^4^	Bootstrapping 95th percentileheritability estimate, %^4^
Insulin^2^	Carbohydrate	49	0	4.0	28.6	5.5	1.11E-23	0.028	5.68
	Protein				7.2	3.1	5.82E-04		
HOMA-IR^2^	Carbohydrate	51	3.2	4.4	27.8	5.2	6.35E-25	0.027	5.97
	Protein				7.5	3.1	6.90E-04		
HOMA-B^3^	n-6 PUFA	39	0	4.0	23.3	5.3	1.68E-22	0.025	5.33
	Protein				1.4	1.7	0.179		

1 h^2^(g), heritability of additive genetic variance; h^2^ (gxe), heritability of GxE interaction; SE, standard error.

2 *P*-values were adjusted for age, sex, study center, kinship, and population structure.

3 *P*-values were adjusted for age, sex, body mass index, study center, kinship, and population structure.

4 95th percentile for the *P*-value and heritability estimate from the 1000× GCTA bootstrap analysis for each trait. In each case, the GCTA GxE *P*-value falls below the 95th percentile indicating that these results are highly unlikely to be obtained merely by chance.

## Discussion

In this study, we have utilized a novel approach to demonstrate the important contribution of GxE interaction to the risk of T2D at the genome-wide level. Using GCTA, we explored the GxE contribution of 15 macronutrients and lifestyle factors to the total phenotypic variance of four T2D-related traits. Our results showed that 25.1% and 24.2% of the heritability of fasting insulin and HOMA-IR could be explained by the GxE interaction of carbohydrate intake with the whole genome, and that 39.0% of the heritability of HOMA-B could be explained by the GxE interaction of n-6 PUFA with the genome. The heritability explained by the main effect of the genome without GxE in the model was only 20.2%, 20.9% and 18.7% for fasting insulin, HOMA-IR and HOMA-B, respectively. For each trait, we selected one environmental factor with the most significant GxE variance contribution and another one with a non-significant GxE variance contribution as a control, and conducted GxE GWAS to illustrate the GCTA results. For insulin and HOMA-IR, carbohydrate intake contributed the most significant GxE and the corresponding GxE GWAS identified 28 and 27 significant SNPs (*P*<1.0E-5), respectively, and these numbers were larger than that identified from the control factor. For HOMA-B, GCTA identified n-6 PUFA to be the most important factor contributing to GxE variance, and 26 significant SNPs were identified through the GxE GWAS for n-6 PUFA, while it was only two significant SNPs for the control dietary factor.

With the maturity of GWAS analysis, understanding the genome-wide variance contribution of GxE interaction to the disease phenotypes, such as T2D-related traits, is becoming a primary interest for researchers. The first GWAS for T2D was published in 2007 [25] and more than 30 GWAS for T2D have been published since then [26]. Although the results produced by these T2D GWAS were intriguing as more than 30 novel T2D loci have been identified, a great number of genetic variants may still be overlooked in the traditional GWAS without the influence of GxE interaction [11]. For example, most of those GWAS-identified SNPs were related to impaired β-cell function, while only a few SNPs were related to insulin resistance [9], [27], [28], suggesting that environmental factors may play an important role in insulin resistance. As indicated in this study, carbohydrate intake contributed a significant GxE variance to the variance of insulin resistance, and GWAS including GxE into the model explained more insulin resistance variance and greatly increased the number of significant SNPs compared with GWAS without GxE (**Table 3**).

Additional evidence supporting a potentially important role for environmental modulation of genetic risk was found in previous population studies. For example, although some of the GWAS-identified T2D loci could be replicated successfully in various populations (e.g., *CDKAL1*, *HHEX*, *IGF2BP2*, *TCF7L2* and *SLC30A8*), more genetic variants have been identified only in some specific populations [26]. T2D risk alleles showed extreme directional differentiation between different populations compared with other common diseases [29]. Different T2D loci and loci frequencies across different populations may reflect the adaptation to the local environments and diets along with human migration [30]. Therefore, the interplay between gene and environment leads to a more complex pathogenesis of T2D and related traits. These hypotheses are strongly supported by a number of recent GxE studies [7], [11], [31], [32]. For example, Qi *et al*. [31] generated a genetic risk score (GRS) using ten GWAS-identified SNPs and observed a significant interaction between the Western dietary pattern and GRS in the Health Professionals Follow-Up Study. The Western dietary pattern was only positively associated with risk of T2D among men with a high GRS, but not with low GRS subjects. Another large meta-analysis of 14 cohort studies [32] revealed that dietary whole-grain intake potentially interacted with one *GCKR* variant (rs780094) for fasting insulin in individuals of European descent. Greater whole-grain intake was associated with a smaller reduction of fasting insulin in individuals with the insulin-raising allele of rs780094, compared to the non-risk allele. Our study provides further evidence of a compelling nature that GxE interactions contribute to the variance of T2D-related traits at the genome-wide level, thereby profoundly influencing the risk of T2D.

In the current study, different interaction patterns were observed for different T2D-related traits. For insulin and HOMA-IR, significant GxE variance contributions of carbohydrate were observed, while for HOMA-B, n-6 PUFA contributed significantly to the GxE interaction with the genome. These findings provided important clues for the further studies relevant to the prevention of T2D through nutritional interventions. For example, n-3 PUFA have been well known for their cardioprotective effects [33], [34] and possible beneficial effects on insulin resistance and T2D [35], [36], however meta-analyses from prospective studies have found overall null association for n-3 PUFA and risk of T2D [37], [38], and opposite trends between Western populations (positive association) and Eastern populations (inverse association). Results from randomized controlled trials of n-3 PUFA on insulin resistance [39] or glycemic traits [40] were also inconsistent. These inconsistencies may be attributed to the GxE interaction as suggested by the present study. Variance of the GxE interaction for n-3: n-6 PUFA ratio accounted for 15.3% heritability of HOMA-IR, while it was 17.4% for fasting insulin. And for fasting glucose, 11.3% heritability of glucose was attributed to the GxE of n-3 PUFA. As the environmental factors were population-specific, different populations may possess different GxE patterns and different disease risk, and these different GxE patterns may contribute to the different response of T2D risk to n-3 PUFA intake among Western and Eastern populations. Therefore, future intervention or cohort studies with regard to n-3 PUFA and T2D and related traits should always take into consideration GxE interactions. In addition to n-3 PUFA, carbohydrate intake showed a crucial role to interact with the whole genome to influence insulin resistance and fasting insulin concentration in the present study, while dietary glycemic load did not show significant GxE on any T2D-related trait. Our previous studies [41], [42] identified *PLIN1* variants that interact with the saturated fatty acid-to-carbohydrate ratio to influence insulin resistance. However, GxE studies that investigate relationships between carbohydrate intake and insulin resistance remain limited [7]. More work is clearly needed to explore the GxE of carbohydrate intake with potential genetic variants for insulin resistance and related traits.

Another finding of interest is the significant GxE variance contribution of n-6 PUFA to HOMA-B. PUFAs, including both n-3 and n-6 families, were suggested to improve insulin sensitivity through incorporation into the cell membrane, and increased membrane fluidity [43]. However, the mechanisms for these effects on β-cell function are less clear. The present study indicated that n-6 PUFA, compared to n-3 PUFA or other dietary factors, had a greater number of interactive relationships with the genome to affect β-cell function, and these interactions are biologically plausible. For example, two SNPs (rs6533014 and rs6533015) showing a significant GxE interaction with n-6 PUFA map near the *NFKB1* gene. NF-kB, an important regulator of expression of genes involved in a variety of biological functions, is involved in the regulation of β-cell function via control of glucose-stimulated insulin secretion [44]. Another example was that eight of those 26 SNPs showing a significant GxE interaction with n-6 PUFA are located in the *FAT3*-*MTNR1B* region (**Table S6**). GWAS have identified several SNPs in this region to be associated with T2D and fasting glucose [3], [26]. Therefore, n-6 PUFA may interact with genetic variants in this region to regulate glucose and β-cell function, thereby affecting T2D risk. However, the precise mechanisms by which n-6 PUFA influences β-cell function via the NF-kB pathway or *FAT3*-*MTNR1B* region, and the function of the identified SNPs warrants further investigation. Nevertheless, these findings provided insight into the extent of the interplay of n-6 PUFA with the genome in regard to β-cell function.

Possible overestimation of genetic and GxE variance may be a limitation of this study, as GOLDN is a family-based population, and causal genetic variants might be captured by pedigree instead of SNPs [6], [45]. Similar dietary and lifestyle factors within a family would also bias the variance estimation. Second, the moderate sample size of the present study only allowed us to estimate GxE variance for each environmental factor separately. In addition, the sum of the heritability explained by the environmental factors was more than 100%; this rose from the high correlations between several of the environmental factors. Third, none of the GCTA results passed the Bonferroni correction (*P*<0.001). Nevertheless, our GxE GWAS confirmed the GCTA results, and a great difference was observed between the significant environmental factor and the control factor for each trait. Fourth, GCTA based on those GxE GWAS-identified SNPs further confirmed the primary GCTA results. Overall, we have shown that adding a GxE interaction into the GWAS model explained a greater degree of heritability for three T2D-related traits than examining genetic effects alone. These results indicate the importance of examining GxE interactions to explain the variance of T2D-related traits. In addition, our results were observed in a European population living in the U.S.A, and may not be applicable to other populations with different genotypes, ancestry, haplotypes, or different cultures and their different lifestyle choices.

In conclusion, we have presented a new approach to demonstrate the important contribution of GxE interaction at the genome-wide level to the heritability of T2D-related traits. In contrast to traditional GWAS, GxE GWAS has the potential to unveil novel genetic variants associated with disease risk, and, importantly, those whose risk is potentially modifiable by lifestyle intervention. The methods presented herein will facilitate a better prediction of T2D and can also be applied to the prediction of other diseases, especially metabolic diseases and cancer for which we have noted many GxE interactions are already known.

## Supporting Information

Figure S1 QQ-plot: Fasting insulin.
(DOCX)

Figure S2 QQ-plot: HOMA-IR.
(DOCX)

Figure S3 QQ-plot: HOMA-B.
(DOCX)

Table S1 Estimation of additive genetic variance and variance of GxE interaction for fasting glucose.
(DOCX)

Table S2 Estimation of additive genetic variance and variance of GxE interaction for fasting insulin.
(DOCX)

Table S3 Estimation of additive genetic variance and variance of GxE interaction for HOMA-IR.
(DOCX)

Table S4 Estimation of additive genetic variance and variance of GxE interaction for HOMA-B.
(DOCX)

Table S5 Estimation of GxE variance for paired environmental factors on T2D traits.
(DOCX)

Table S6 SNP list identified from GxE GWAS for diabetes traits in GOLDN population.
(XLSX)
