# Supplementary material for: Genome-Wide Contribution of Genotype by Environment Interaction to Variation of Diabetes-Related Traits
Source: PLoS One. 2013 Oct 28;8(10):e77442. doi: 10.1371/journal.pone.0077442 (PMC3810463; doi:10.1371/journal.pone.0077442)
Supplement: Figure S1 — QQ-plot: Fasting insulin. (DOCX) [file pone.0077442.s001.docx]

**Figure S1 QQ-plot: Fasting insulin**
