# Supplementary material for: Genome-Wide Contribution of Genotype by Environment Interaction to Variation of Diabetes-Related Traits
Source: PLoS One. 2013 Oct 28;8(10):e77442. doi: 10.1371/journal.pone.0077442 (PMC3810463; doi:10.1371/journal.pone.0077442)
Supplement: Figure S2 — QQ-plot: HOMA-IR. (DOCX) [file pone.0077442.s002.docx]

**Figure S2 QQ-plot: HOMA-IR**
