# Supplementary material for: Genome-Wide Contribution of Genotype by Environment Interaction to Variation of Diabetes-Related Traits
Source: PLoS One. 2013 Oct 28;8(10):e77442. doi: 10.1371/journal.pone.0077442 (PMC3810463; doi:10.1371/journal.pone.0077442)
Supplement: Table S1 — Estimation of additive genetic variance and variance of GxE interaction for fasting glucose. (DOCX) [file pone.0077442.s004.docx]

**Table S1 Estimation of additive genetic variance and variance of GxE interaction for fasting glucose^1^**

| E factor | *P*-value (gxe) | Vg | SE | Vgxe | SE | h^2^ (g), % | SE | h^2^ (gxe), % | SE | h^2^ (g+gxe), % |
| --- | --- | --- | --- | --- | --- | --- | --- | --- | --- | --- |
| Glycemic load | 0.434 | 0.723 | 0.324 | 0.095 | 0.549 | 18.8 | 8.2 | 2.5 | 14.3 | 21.3 |
| Protein | 0.500 | 0.760 | 0.328 | 0 | 0.490 | 19.9 | 8.4 | 0 | 12.8 | 19.9 |
| Total fat | 0.500 | 0.563 | 0.309 | 0 | 0.539 | 14.8 | 8.0 | 0 | 14.1 | 14.8 |
| Saturated fat | 0.500 | 0.600 | 0.319 | 0 | 0.524 | 15.7 | 8.3 | 0 | 13.7 | 15.7 |
| MUFA | 0.500 | 0.574 | 0.314 | 0 | 0.537 | 15.0 | 8.1 | 0 | 14.1 | 15.0 |
| PUFA | 0.302 | 0.704 | 0.323 | 0.264 | 0.523 | 18.3 | 8.2 | 6.8 | 13.6 | 25.1 |
| n-3 PUFA | 0.173 | 0.671 | 0.324 | 0.435 | 0.501 | 17.5 | 8.3 | 11.3 | 13.0 | 28.8 |
| n-6 PUFA | 0.269 | 0.690 | 0.324 | 0.310 | 0.519 | 17.9 | 8.2 | 8.1 | 13.5 | 26.0 |
| n-3: n-6 PUFA | 0.437 | 0.712 | 0.334 | 0.081 | 0.509 | 18.6 | 8.5 | 2.1 | 13.3 | 20.7 |
| Carbohydrate | 0.500 | 0.819 | 0.323 | 0 | 0.479 | 21.5 | 8.2 | 0 | 12.6 | 21.5 |
| Alcohol use | 0.212 | 0.613 | 0.373 | 0.359 | 0.484 | 15.9 | 9.5 | 9.3 | 12.5 | 25.2 |
| Trans fat | 0.500 | 0.676 | 0.315 | 0 | 0.562 | 17.6 | 8.0 | 0 | 14.6 | 17.6 |
| Fiber | 0.310 | 0.704 | 0.328 | 0.270 | 0.533 | 18.5 | 8.4 | 7.1 | 14.0 | 25.5 |
| Physical activity | 0.500 | 0.703 | 0.331 | 0 | 0.514 | 18.4 | 8.5 | 0.0 | 13.4 | 18.4 |
| Smoking status | 0.500 | 0.776 | 0.442 | 0 | 0.493 | 20.2 | 11.4 | 0.0 | 12.9 | 20.2 |

^1^ Without GxE: phenotypic variance Vp=3.83 (0.19), Vg=0.759 (0.296), h^2^ (g)=19.8% (7.5%), *P*-value (g)=0.002. *P*-value (gxe) of GxE interaction was adjusted for age, sex, study center, kinship, and population structure.Vg, additive genetic variance; Vgxe,variance contributed by GxE interaction; SE, standard error; h^2^ (g), heritability; h^2^ (g+gxe)=total heritability.
