# Supplementary material for: Genome-Wide Contribution of Genotype by Environment Interaction to Variation of Diabetes-Related Traits
Source: PLoS One. 2013 Oct 28;8(10):e77442. doi: 10.1371/journal.pone.0077442 (PMC3810463; doi:10.1371/journal.pone.0077442)
Supplement: Table S2 — Estimation of additive genetic variance and variance of GxE interaction for fasting insulin. (DOCX) [file pone.0077442.s005.docx]

**Table S2 Estimation of additive genetic variance and variance of GxE interaction for fasting insulin^1^**

| E factor | *P*-value (gxe) | Vg | SE | Vgxe | SE | h^2^ (g), % | SE | h^2^ (gxe), % | SE | h^2^ (g+gxe), % |
| --- | --- | --- | --- | --- | --- | --- | --- | --- | --- | --- |
| Glycemic load | 0.369 | 0.00061 | 0.00030 | 0.00017 | 0.00049 | 17.6 | 8.4 | 4.8 | 14.2 | 22.5 |
| Protein | 0.500 | 0.00058 | 0.00030 | 0 | 0.00050 | 16.4 | 8.5 | 0 | 14.4 | 16.5 |
| Total fat | 0.125 | 0.00055 | 0.00031 | 0.00057 | 0.00050 | 15.7 | 8.7 | 16.2 | 14.2 | 31.9 |
| Saturated fat | 0.312 | 0.00065 | 0.00032 | 0.00024 | 0.00049 | 18.5 | 8.9 | 6.9 | 13.9 | 25.5 |
| MUFA | 0.358 | 0.00067 | 0.00030 | 0.00019 | 0.00051 | 18.9 | 8.3 | 5.5 | 14.4 | 24.4 |
| PUFA | 0.500 | 0.00065 | 0.00030 | 0 | 0.00049 | 18.3 | 8.2 | 0 | 13.9 | 18.3 |
| n-3 PUFA | 0.344 | 0.00064 | 0.00031 | 0.00020 | 0.00050 | 18.2 | 8.5 | 5.8 | 14.1 | 23.9 |
| n-6 PUFA | 0.500 | 0.00066 | 0.00030 | 0 | 0.00049 | 18.7 | 8.4 | 0 | 14.0 | 18.7 |
| n-3: n-6 PUFA | 0.112 | 0.00056 | 0.00031 | 0.00062 | 0.00051 | 15.7 | 8.6 | 17.4 | 14.2 | 33.1 |
| **Carbohydrate** | **0.032** | **0.00048** | **0.00031** | **0.00089** | **0.00050** | **13.6** | **8.6** | **25.1** | **14.0** | **38.7** |
| Alcohol use | 0.500 | 0.00065 | 0.00034 | 0 | 0.00044 | 19.0 | 9.8 | 0 | 12.7 | 19.0 |
| Trans fat | 0.500 | 0.00056 | 0.00029 | 0 | 0.00052 | 16.1 | 8.2 | 0 | 14.8 | 16.1 |
| Fiber | 0.314 | 0.00068 | 0.00031 | 0.00023 | 0.00048 | 19.5 | 8.6 | 6.7 | 13.7 | 26.2 |
| Physical activity | 0.367 | 0.00063 | 0.00031 | 0.00016 | 0.00048 | 17.9 | 8.7 | 4.7 | 13.6 | 22.6 |
| Smoking status | 0.142 | 0.00038 | 0.00043 | 0.00054 | 0.00052 | 10.9 | 12.2 | 15.5 | 14.9 | 26.4 |

^1^ Without GxE: phenotypic variance Vp=0.0035, Vg=0.0007, h^2^ (g)=20.2% (7.5%), *P*-value (g)=0.002. *P*-value (gxe) of GxE interaction was adjusted for age, sex, study center, kinship, and population structure.Vg=additive genetic variance, Vgxe=variance contributed by GxE interaction, SE=standard error, h^2^ (g)=heritability, h^2^ (g+gxe)=total heritability.
