# Supplementary material for: Genome-Wide Contribution of Genotype by Environment Interaction to Variation of Diabetes-Related Traits
Source: PLoS One. 2013 Oct 28;8(10):e77442. doi: 10.1371/journal.pone.0077442 (PMC3810463; doi:10.1371/journal.pone.0077442)
Supplement: Table S3 — Estimation of additive genetic variance and variance of GxE interaction for HOMA-IR. (DOCX) [file pone.0077442.s006.docx]

**Table S3 Estimation of additive genetic variance and variance of GxE interaction for HOMA-IR^1^**

| E factor | *P*-value (gxe) | Vg | SE | Vgxe | SE | h^2^ (g), % | SE | h^2^ (gxe), % | SE | h^2^ (g+gxe), % |
| --- | --- | --- | --- | --- | --- | --- | --- | --- | --- | --- |
| Glycemic load | 0.398 | 0.0016 | 0.0007 | 0.0003 | 0.0012 | 18.5 | 8.4 | 3.7 | 14.1 | 22.1 |
| Protein | 0.500 | 0.0015 | 0.0008 | 0 | 0.0012 | 17.7 | 8.6 | 0 | 14.3 | 17.7 |
| Total fat | 0.159 | 0.0015 | 0.0008 | 0.0012 | 0.0012 | 17.1 | 8.6 | 14.0 | 14.1 | 31.0 |
| Saturated fat | 0.366 | 0.0017 | 0.0008 | 0.0004 | 0.0012 | 20.0 | 8.8 | 4.8 | 13.7 | 24.8 |
| MUFA | 0.422 | 0.0017 | 0.0007 | 0.0003 | 0.0012 | 20.1 | 8.3 | 3.0 | 14.3 | 23.1 |
| PUFA | 0.500 | 0.0016 | 0.0007 | 0 | 0.0012 | 18.8 | 8.2 | 0 | 13.8 | 18.8 |
| n-3 PUFA | 0.331 | 0.0016 | 0.0008 | 0.0005 | 0.0012 | 18.7 | 8.5 | 6.2 | 14.0 | 24.9 |
| n-6 PUFA | 0.500 | 0.0017 | 0.0007 | 0 | 0.0012 | 19.1 | 8.3 | 0 | 14.0 | 19.1 |
| n-3: n-6 PUFA | 0.140 | 0.0015 | 0.0008 | 0.0013 | 0.0012 | 16.7 | 8.6 | 15.3 | 14.1 | 32.0 |
| **Carbohydrate** | **0.035** | **0.0013** | **0.0008** | **0.0021** | **0.0012** | **14.5** | **8.6** | **24.2** | **13.9** | **38.7** |
| Alcohol use | 0.500 | 0.0017 | 0.0008 | 0 | 0.0011 | 19.6 | 9.7 | 0 | 12.6 | 19.6 |
| Trans fat | 0.500 | 0.0014 | 0.0007 | 0 | 0.0013 | 16.4 | 8.2 | 0 | 14.7 | 16.4 |
| Fiber | 0.313 | 0.0017 | 0.0008 | 0.0006 | 0.0012 | 20.1 | 8.6 | 6.7 | 13.7 | 26.8 |
| Physical activity | 0.363 | 0.0016 | 0.0008 | 0.0004 | 0.0012 | 18.5 | 8.7 | 4.8 | 13.6 | 23.3 |
| Smoking status | 0.152 | 0.0011 | 0.0011 | 0.0013 | 0.0013 | 12.1 | 12.2 | 14.8 | 14.8 | 26.9 |

^1^ Without GxE: phenotypic variance Vp=0.0087, Vg=0.0018, h^2^ (g)=20.9% (7.5%), *P*-value (g)=0.001. *P*-value (gxe) of GxE interaction was adjusted for age, sex, study center, kinship, and population structure. Vg=additive genetic variance, Vgxe=variance contributed by GxE interaction, SE=standard error, h^2^ (g)=heritability, h^2^ (g+gxe)=total heritability.
