# Supplementary material for: Genome-Wide Contribution of Genotype by Environment Interaction to Variation of Diabetes-Related Traits
Source: PLoS One. 2013 Oct 28;8(10):e77442. doi: 10.1371/journal.pone.0077442 (PMC3810463; doi:10.1371/journal.pone.0077442)
Supplement: Table S4 — Estimation of additive genetic variance and variance of GxE interaction for HOMA-B. (DOCX) [file pone.0077442.s007.docx]

**Table S4 Estimation of additive genetic variance and variance of GxE interaction for HOMA-B^1^**

| E factor | *P*-value (gxe) | Vg | SE | Vgxe | SE | h^2^ (g), % | SE | h^2^ (gxe), % | SE | h^2^ (g+gxe), % |
| --- | --- | --- | --- | --- | --- | --- | --- | --- | --- | --- |
| Glycemic load | 0.391 | 202.8 | 103.3 | 48.1 | 167.1 | 17.5 | 8.7 | 4.1 | 14.4 | 21.6 |
| Protein | 0.500 | 189.6 | 102.6 | 0 | 166.4 | 16.2 | 8.7 | 0 | 14.2 | 16.2 |
| Total fat | 0.066 | 166.6 | 103.8 | 233.4 | 164.7 | 14.2 | 8.7 | 19.8 | 13.9 | 34.0 |
| Saturated fat | 0.500 | 210.3 | 102.2 | 0 | 155.0 | 17.9 | 8.5 | 0 | 13.2 | 17.9 |
| MUFA | 0.068 | 172.1 | 102.3 | 238.3 | 166.9 | 14.7 | 8.6 | 20.3 | 14.1 | 35.0 |
| **PUFA** | **0.016** | **148.7** | **102.7** | **370.0** | **175.4** | **12.6** | **8.6** | **31.4** | **14.6** | **44.0** |
| n-3 PUFA | 0.215 | 197.9 | 102.4 | 128.9 | 165.4 | 16.8 | 8.5 | 11.0 | 14.0 | 27.8 |
| **n-6 PUFA** | **0.005** | **105.5** | **104.5** | **459.8** | **180.4** | **8.9** | **8.8** | **39.0** | **14.9** | **48.0** |
| n-3: n-6 PUFA | 0.156 | 175.0 | 106.0 | 165.5 | 165.7 | 14.9 | 8.9 | 14.1 | 14.0 | 28.9 |
| Carbohydrate | 0.238 | 215.7 | 99.7 | 120.8 | 163.3 | 18.7 | 8.4 | 10.5 | 14.1 | 29.1 |
| Alcohol use | 0.500 | 222.3 | 114.4 | 0 | 147.8 | 19.3 | 9.8 | 0 | 12.8 | 19.3 |
| Trans fat | 0.219 | 189.5 | 101.7 | 152.2 | 179.7 | 16.1 | 8.5 | 12.9 | 15.2 | 29.0 |
| Fiber | 0.500 | 208.7 | 103.0 | 0 | 166.8 | 17.9 | 8.6 | 0 | 14.3 | 17.9 |
| Physical activity | 0.500 | 189.5 | 104.0 | 0 | 153.6 | 16.3 | 8.8 | 0 | 13.2 | 16.3 |
| **Smoking status** | **0.055** | **49.0** | **145.1** | **255.7** | **174.9** | **4.2** | **12.5** | **22.0** | **14.9** | **26.2** |

^1^ Without GxE: phenotypic variance Vp=1170.4 (59.0), Vg=219.2 (92.3), h2 (g)=18.7% (7.7%), *P*-value (g)=0.005. *P*-value (gxe) of GxE interaction was adjusted for age, sex, body mass index, study center, kinship, and population structure. Vg=additive genetic variance, Vgxe=variance contributed by GxE interaction, SE=standard error, h^2^ (g)=heritability, h^2^ (g+gxe)=total heritability.
