# Supplementary material for: Genome-Wide Contribution of Genotype by Environment Interaction to Variation of Diabetes-Related Traits
Source: PLoS One. 2013 Oct 28;8(10):e77442. doi: 10.1371/journal.pone.0077442 (PMC3810463; doi:10.1371/journal.pone.0077442)
Supplement: Table S5 — Estimation of GxE variance for paired environmental factors on T2D traits. (DOCX) [file pone.0077442.s008.docx]

**Table S5 Estimation of GxE variance for paired environmental factors on T2D traits^1^**

| Trait | E factor | h^2^ (g) | SE | h^2^ (gxe) | SE | *P*-value |
| --- | --- | --- | --- | --- | --- | --- |
| Insulin ^2^ | Carbohydrate | 11.1 | 9.4 | 22.4 | 14.2 | 0.056 |
|  | n-3: n-6 PUFA ratio | |  | 13.1 | 13.9 | 0.176 |
|  |  |  |  |  |  |  |
|  | Carbohydrate | 11.9 | 9.2 | 19.8 | 14.5 | 0.078 |
|  | Total fat |  |  | 11.8 | 14.6 | 0.205 |
|  |  |  |  |  |  |  |
|  | Carbohydrate | 4.9 | 12.6 | 23.6 | 13.9 | 0.039 |
|  | Smoking status |  |  | 14.9 | 14.9 | 0.150 |
|  |  |  |  |  |  |  |
| HOMA-IR ^2^ | Carbohydrate | 12.1 | 9.3 | 21.6 | 14.1 | 0.060 |
|  | n-3: n-6 PUFA ratio | |  | 11.6 | 13.8 | 0.203 |
|  |  |  |  |  |  |  |
|  | Carbohydrate | 13.2 | 9.2 | 19.6 | 14.4 | 0.077 |
|  | Total fat |  |  | 9.4 | 14.4 | 0.254 |
|  |  |  |  |  |  |  |
|  | Carbohydrate | 6.2 | 12.6 | 22.6 | 13.8 | 0.044 |
|  | Smoking status |  |  | 14.4 | 14.8 | 0.157 |
|  |  |  |  |  |  |  |
| HOMA-B ^3^ | n-6 PUFA | 5.9 | 8.7 | 45.5 | 34.1 | 0.147 |
|  | PUFA |  |  | 0.0 | 33.3 | 0.500 |
|  |  |  |  |  |  |  |
|  | n-6 PUFA | 5.6 | 9.4 | 37.3 | 15.0 | 0.007 |
|  | MUFA |  |  | 16.6 | 14.0 | 0.110 |
|  |  |  |  |  |  |  |
|  | n-6 PUFA | 7.7 | 9.3 | 35.8 | 15.6 | 0.014 |
|  | Total fat |  |  | 8.8 | 14.0 | 0.261 |
|  |  |  |  |  |  |  |
|  | n-6 PUFA | 0.0 | 12.9 | 33.4 | 14.6 | 0.012 |
|  | Smoking status |  |  | 19.9 | 14.6 | 0.066 |

^1^ SE, standard error; h^2^ (g), heritability of additive genetic variance; h^2^ (gxe), heritability of GxE interaction.

^2^ *P*-values were adjusted for age, sex, study center, kinship, and population structure.

^3^ *P*-values were adjusted for age, sex, body mass index, study center, kinship, and population structure.
